# Baseline respiratory system compliance and its decline during general anesthesia for surgery: A retrospective observational study

**DOI:** 10.1097/MD.0000000000042845

**Published:** 2025-06-13

**Authors:** Katsuya Tanaka, Koji Hosokawa, Kayo Ishihara, Yukiko Yamazaki, Yuka Matsuki, Kenji Shigemi

**Affiliations:** a Department of Anesthesiology and Reanimatology, Faculty of Medical Sciences, University of Fukui, Fukui, Japan; b Department of Anesthesiology, Municipal Tsuruga Hospital, Fukui, Japan; c Department of Intensive Care, University of Fukui Hospital, Fukui, Japan; d Department of Intensive Care, Rakuwakai Otowa Hospital, Kyoto, Japan; e Department of Surgery, Tokyo Metropolitan Otsuka Hospital, Tokyo, Japan; f Faculty of Medicine, University of Fukui, Fukui, Japan.

**Keywords:** lung compliance, outcome, respiratory mechanics, retrospective studies

## Abstract

Maintaining respiratory system compliance (Crs) is a clue for lung recruitment strategies during surgery; however, patients’ baseline Crs values have not been adequately addressed. We studied the association of low Crs at the induction of anesthesia (iCrs) with limited intraoperative respiratory management. We conducted a secondary analysis of retrospective study of consecutive surgical patients on ventilators between 2019 and 2020. The intra- and postoperative respiratory-related parameters were compared between the low iCrs group (<25% of the distribution) and others. Primary outcome was postoperative respiratory support related with iCrs and intraoperative Crs decline. Multivariate analysis and other root-cause analysis were performed to evaluate the relationships between low iCrs and outcomes. A total of 5568 patients were included and classified into either the low iCrs group (<43 mL/cmH_2_O, n = 1392) or the other group (n = 4176). The age was older and both the duration of surgery and the duration of anesthesia were shorter in the low iCrs group than in the other groups. Unexpectedly, the low iCrs group was associated with a smaller decrease in hourly changes in Crs (−1.4% [−11.3% to 11.2%] vs −13.0% [−21.3% to −5.3%], *P* < .001). Despite the smaller decrease in Crs, the low iCrs group showed a higher incidence of > 3 days of postoperative oxygen therapy (adjusted OR 1.60 (1.29–1.97), *P* < .001) and > 3 days of mechanical ventilation following surgery (adjusted OR, 1.55 (1.02–2.36), *P* = .039) than the other group. The significances in postoperative therapies were exaggerated in emergency surgery. The low iCrs patients showed a smaller decline in hourly changes in Crs and longer postoperative respiratory supports. Not only the decrease in Crs during surgery but also the initial Crs value warrant the intra- and postoperative respiratory management.

## 1. Introduction

Respiratory system compliance (Crs) depends on the mechanical stiffness of the lungs and chest wall. It influences the dynamics of respiration during mechanical ventilation.^[1]^ Crs is calculated as the tidal volume divided by the difference between plateau pressure and positive end-expiratory pressure (PEEP). For surgical patients under mechanical ventilation, the decrease in Crs during surgery suggests atelectasis or closing lung capacity.^[2–4]^ Thus, maintaining Crs using the lung recruitment maneuver or PEEP successfully reduces the risk of postoperative complications.^[5,6]^

Intraoperative monitoring of Crs is undoubtedly helpful.^[7,8]^ However, we often find that patients with originally low Crs do not achieve the planned Crs. Additionally, the surgical procedure or abdominal pressure due to laparotomy would decrease Crs and supposedly limit the Crs management in patients with initially low Crs. We hypothesize that patients with low Crs at the induction of anesthesia (iCrs) may be associated with poor intraoperative Crs management and postoperative outcomes. In this study, we investigated the relationship of patients with low iCrs with intraoperative changes in Crs. Additionally, we investigated the association between low iCrs and other clinical outcomes, including the duration of postoperative oxygen therapy. Then, root-cause analyses of observed discrepancies were conducted.

## 2. Methods

We conducted a secondary retrospective observational study using local electronic medical records and the anesthesia chart database.^[9,10]^ The research involving human data was performed in accordance with the Declaration of Helsinki. Ethical approval for this study (#20210023) was provided by the Research Ethics Committee of the University of Fukui (Fukui, Japan; Chairperson, Prof Masaru Inatani) on May 17, 2021. The requirement for written informed consent from the participants was waived by the Research Ethics Committee of the University of Fukui because their patients were basically informed that the existing clinical data were used for research and publication. However, as an opt-out policy, the study information was cited on the hospital website, and the participants were allowed to deny their inclusion in the study via direct contact with the researchers. The study followed the Strengthening the Reporting of Observational Studies in Epidemiology (STROBE) Statement.^[11]^ Anesthesia induction and respiratory settings were shown in the Supplemental document (Supplemental Digital Content, https://links.lww.com/MD/P169).

### 2.1. Database

The generation of database was described previously.^[9,10]^ Briefly, using the GAIA anesthesia chart system (Nihon Koden, Tokyo, Japan) between January 1, 2019 and December 31, 2020, the patient characteristics were conjugated with respiratory parameters measured on an Aisys CS 2 (GE Healthcare, Chicago) every minute during anesthesia and then, we electronically conjugated the patients’ data retrieved from the hospital electronic medical records, which was operated by IBM (Tokyo, Japan).

### 2.2. Respiratory parameters

Crs was displayed on Aisys CS 2 using manufacture-driven calculations. The normal rage of Crs is 45 to 70 mL/cmH_2_O in general adult patients.^[3]^ Other respiratory parameters were also measured, regardless of the respiratory mode. These values were stored on the GAIA anesthesia chart system and extracted every minute during anesthesia. iCrs (Crs just after tracheal intubation while the induction of anesthesia was performed) was the mean of 9 consecutive min values after tracheal intubation. The 1 hourly Crs values were the means of the Crs values close to each time-point. Crs before extubation was the mean of 7 consecutive min values before extubation. The hourly changes in Crs were calculated.

### 2.3. Patient

All consecutive patients who underwent respiratory management were included in the study. Patients with missing iCrs values were also excluded.

### 2.4. Outcomes

The primary outcome was the intraoperative hourly change in Crs during surgery between the low iCrs group and the others. The secondary outcome was the difference in postoperative respiratory management, including oxygen therapy, mechanical ventilation, intensive care unit (ICU) stay, and mortality, between the low iCrs group and the others.

### 2.5. Sample size

For sample size, 505 patients were estimated to be required when the α was 0.05, and a detection power of 80% was assumed for a 5% increase of the hourly Crs change in the 20% of standard deviation.

### 2.6. Statistical analysis

We defined low iCrs as the lower 25% of the distribution of Crs. From previous investigation,^[9,10]^ the cutoff of iCrs was close to the physiologically significant threshold. Moreover, Crs was treated as a binary parameter since it was not linearly correlated to other results. The outcome measures were compared between the low iCrs group and the others using the Wilcoxon test. The correlation between 2 parameters (iCrs and hourly change in Crs) was drawn graphically. For the relationship between the 2 values, a second-order approximate curve was drawn, and a 95% confidence interval was added. The duration of postoperative oxygen therapy was compared between the 2 groups using Kaplan–Meier curves and tested using the log-rank test. The number of days of postoperative oxygen administration was classified by an arbitrarily set number of days (3 days), and odds ratios between the 2 groups were calculated using logistic regression analysis, adjusting for age, ASA physical status, surgical categories, and duration of anesthesia. The same analyses were conducted for other parameters.

Sensitivity analysis was conducted according to the textbook by Lash.^[12]^ Assuming that the effect of unknown confounders varied from an odds ratio of 0.1 to 10, and that the frequency of unknown confounders varied from equal to twice as much, adjusted odds ratios were calculated. Additionally, sensitivity analysis was conducted for different iCrs categories. For root-cause analysis and subgroup analysis, subgroups of emergency patients were analyzed separately.

The combined databases were managed using Microsoft Excel (Microsoft, Redmond). All graphs and statistical analyses were completed using JMP 17 Pro software (SAS Institute Inc., Cary).

## 3. Results

### 3.1. Patient characteristics

A total of 5568 patients were included. The top 3 surgical categories were abdominal surgery (32.5%), head and neck surgery (26.9%), and orthopedic surgery (21.7%) (Table 1). Laparoscopic procedures were performed in 18.0% of the patients.

**Table 1:** Patient characteristics and parameters during anesthesia.

	All case (n = 5568)	Crs value at induction of anesthesia		
		<43 mL/cmH_2_O (n = 1392)	≥43 mL/cmH_2_O (n = 4176)	*P*-value
Age (yr)	65 (48–74)	70 (56–79)	62 (46–72)	.001
Male:female	50.5%:49.5%	30.9%:69.1%	57.1%:42.9%	.001
Body mass index (kg · m^-2^)	23 (21–26)	24 (21–27)	23 (20–25)	.001
ASA physical status				
1, 1E	11.4%–1.3%	7.0%–1.0%	12.8%–1.5%	.001
2, 2E	58.1%–4.1%	48.5%–5.3%	61.2%–3.7%	
3, 3E	21.7%–3.4%	30.9%–7.0%	18.5%–2.2%	
≥ 4, >4E	0.0%–0.1%	0.0%–0.2%	0.0%–0.0%	
Emergency hospital admission	14.8%	20.5%	12.9%	.001
Elective surgery	91.1%	86.4%	92.7%	.001
Surgical category				
Abdominal	32.5%	30.2%	33.2%	.001
Head, neck	26.9%	23.1%	28.1%	
Extremity, spine	21.7%	21.5%	21.8%	
Chest, cardiovascular	10.4%	16.7%	8.3%	
Other	8.6%	8.5%	8.6%	
Laparoscopic procedure	18.0%	14.1%	19.4%	.001
One-lung ventilation	7.1%	12.6%	5.3%	.001
Duration (min)				
From start of anesthesia to intubation	9 (8–12)	9 (8–12)	9 (8–12)	.951
Mechanical ventilation	232 (156–345)	224 (148–336)	234 (160–348)	.009
Surgery	162 (87–270)	144 (82–252)	166 (88–276)	.001
Laparoscopy (in applied cases)	120 (71–210)	117 (69–214)	122 (72–210)	.889
Anesthesia	264 (186–396)	252 (180–375)	268 (188–400)	.001
Mode of respiration				
PCV–VG	94.4%	90.2%	95.8%	.001
PCV	3.0%	5.6%	2.2%	
Other	2.6%	4.3%	2.0%	

Values present median (interquartile range).

ASA = American Society of Anesthesiologists, Crs = respiratory system compliance, PCV = pressure control ventilation, VG = volume guarantee.

### 3.2. Low iCrs and respiratory management during anesthesia

The respiratory parameters during anesthesia are shown in Table S1 (Supplemental Digital Content, https://links.lww.com/MD/P169). The iCrs was 52 (43–63) mL/cmH_2_O. The Crs during surgery decreased, and the hourly change in Crs was −10.8% (−19.4% to −1.6%). This decline in Crs during anesthesia was significant in patients who underwent laparoscopic surgery (−20.3% [−28.7% to −12.2%] vs −9.1% [−16.6% to 0.2%], *P* < .001), with an anesthesia duration >6 hours (−14.0% [−22.2% to −4.6%] vs −10.3% [−18.7% to −1.1%], *P* < .001), and in the prone position (−17.2% [−25.8% to −8.3%] vs −10.3% [−18.6% to −1.2%], *P* < .001).

**Figure 1.**
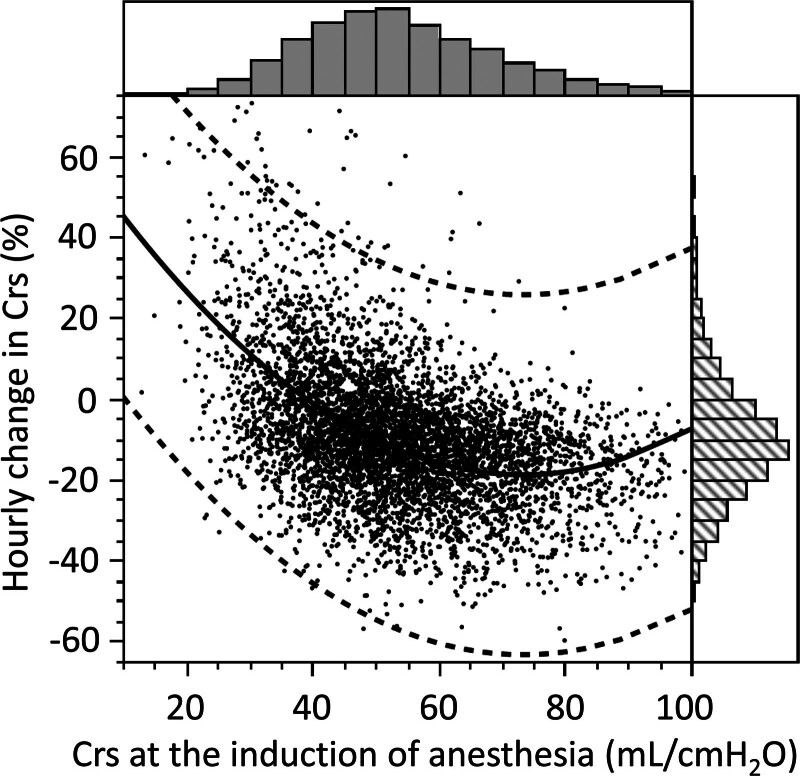
Relationship between initial respiratory system compliance and its hourly changes during anesthesia. A dot represents 1 participant. The smooth spline curve (bold line) and its 95% confidence intervals (dashed lines) are depicted. Histograms were added to upper and right sides of the graph. Crs = respiratory system compliance.

Patients were classified into the low iCrs group (< 43 mL/cmH_2_O, n = 1392) or the other (n = 4176) based on proportion of distribution (Fig. 1). Both the duration of surgery and duration of anesthesia were shorter in the low iCrs group than in the other groups (Table 1). The ventilator mode was almost volume-guaranteed pressure control ventilation (94.4%). From the real-world data and non-protocolized ventilator settings, the tidal volume in the low iCrs group was approximately 10% less than that in the other groups (6.7 [5.9−7.4] mL/kg vs 7.0 [6.4−7.6] mL/kg). The PEEP was similar between the groups.

Interestingly, the decrease in the hourly change in Crs was smaller in the low iCrs group than in the other groups (Table 1). iCrs was negatively correlated with the hourly change in Crs (Fig. 1). The time course changes in the 2 groups are shown in Table S1, Supplemental Digital Content, https://links.lww.com/MD/P169. The rate of change in Crs from intubation to extubation was lower in the low iCrs group than in the others (0.7% [−12.5% to 16.0%] vs −13.0% [−23.7% to −2.5%], *P* < .001).

### 3.3. Low iCrs and postoperative outcomes

The duration of postoperative oxygen therapy was significantly longer in the low iCrs group than in the other groups (Figure S1 and Table S2, Supplemental Digital Content, https://links.lww.com/MD/P169). The median duration of oxygen therapy did not differ clinically (2 [1–2] days vs 2 [1–2] days, *P* < .001; Table S2, Supplemental Digital Content, https://links.lww.com/MD/P169); however, the rate of prolonged oxygen therapy (≥3 days) was higher in the low iCrs group than in the others (adjusted OR, 1.60 [1.29–1.97], *P* < .001; Table S3, Supplemental Digital Content, https://links.lww.com/MD/P169).

The number of patients who needed mechanical ventilation during the 28-day postoperative period was significantly larger in the low iCrs group than in the other groups (adjusted OR, 1.87 [1.33–2.63], *P* < .001; Table S3, Supplemental Digital Content, https://links.lww.com/MD/P169), while there was no clinically significant difference in the median number of days of required mechanical ventilation during the 28-day postoperative period in the examined patients (4 [2–9] days vs 4 [2–7] days, *P* = .078; Table S2, Supplemental Digital Content, https://links.lww.com/MD/P169) or ventilator-free days at 28 days after surgery (28 [28–28] days vs 28 [28–28] days, *P* < .001). However, the rate of mechanical ventilation following surgery (≥4 days) was significantly higher in the low iCrs group than in the other groups (adjusted OR, 1.55 [1.02–2.36], *P* = .039; Table S3, Supplemental Digital Content, https://links.lww.com/MD/P169). The rate of postoperative admission to the ICU was similar (adjusted OR, 1.30 [0.98–1.73]. *P* = .067; Table S3, Supplemental Digital Content, https://links.lww.com/MD/P169). The length of ICU stay was longer (4 [2–8] days vs 3 [2–6] days, *P* < .001; Table S2, Supplemental Digital Content, https://links.lww.com/MD/P169) and the length of hospital stay was longer in the low iCrs group than in the other groups (15 [9–23] days vs 12 [8–21] days, *P* < .001; Table S2, Supplemental Digital Content, https://links.lww.com/MD/P169). In-hospital mortality was similar (Table S2 and S3, Supplemental Digital Content, https://links.lww.com/MD/P169).

### 3.4. Sensitivity analysis

Sensitivity analysis revealed that the adjusted Mantel–Haenszel odds ratio for prolonged oxygen therapy (≥3 days) varied from 2.28 to 5.91, when the effect of unknown confounders varied from an odds ratio of 0.1 to 10, and when the frequency of unknown confounders varied from half to twice as high.

### 3.5. Root-cause analysis

**Figure 2.**
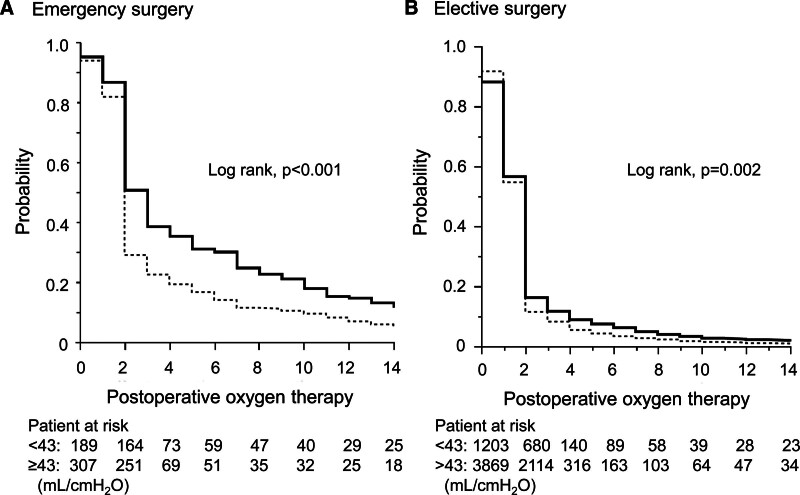
Comparison of the duration of postoperative oxygen therapy between the 2 compliance groups. Low iCrs was defined as Crs < 43 mL/cmH_2_O at the induction of anesthesia. The probability of oxygen therapy is shown in the low iCrs group (bold line) and the other group (dashed lines). The Kaplan–Meier curves revealed that the groups were significantly different (log rank, *P* < .001 for emergency surgery [A] and *P* = .002 in elective surgery [B]). Crs = respiratory system compliance.

This significant difference was exaggerated in emergency surgery and almost disappeared in elective surgery (Fig. 2).

## 4. Discussion

The secondary analysis of a retrospective database-based study showed that patients with low iCrs had smaller hourly decreases in Crs during surgery and a smaller decrease in Crs at extubation than other patients. As expected, the low iCrs group had a longer duration of postoperative oxygen therapy and a higher chance of longer duration of mechanical ventilation. Additionally, we found that in the emergency surgery subgroup, there was a significant association between low iCrs and a longer duration of postoperative oxygen therapy.

The original Crs values of the included surgical patients were measured after a few minutes of preanesthesia preoxygenation, mask ventilation, and apneic intubation procedures. In contrast, the values at that time-point were not affected by body position, abdominal pressure due to laparoscopy, or 1-lung ventilation. Except for patients with interstitial pneumonia, tracheal intubation was performed after oxygenation at F_I_O_2_ 1.0, suggesting that atelectasis would occur.^[13]^ Because of the retrospective nature of the study, it was unclear whether the patient underwent a recruitment procedure after completion of intubation.^[14]^ It would be of scientific interest to determine the frequency of atelectasis at the time of measurement in the present study population.^[15]^ If atelectasis formed more rapidly within a short period in the low iCrs group than in the others, no further atelectasis occurred during subsequent ventilator management. This early atelectasis hypothesis explains the lower rate of Crs decline in the low iCrs group. A smaller tidal volume with higher driving pressure was selected for the low iCrs group under comparable PEEP conditions. These respiratory management methods cannot satisfactorily explain the smaller Crs decrease in the low iCrs group than in the other group. Moreover, the management resulting in a small decrease in Crs was not in line with previous studies showing that decreases in Crs were related to poor postoperative respiratory-related outcomes.^[3,8]^ However, poor postoperative outcomes were likely in the low iCrs group because this group included more vulnerable patients.

Moreover, poor postoperative outcomes in the low iCrs group were more likely to be observed in patients who underwent emergency surgery. Since preoperative assessment has been performed for elective surgical patients, there might have been changes in the anesthesiologist’s respiratory management, such as a more cautious approach.^[4]^ However, in emergency surgery, respiratory-related preoperative management, such as preoperative smoking cessation and asthma treatment, cannot be performed. Patients undergoing emergency surgery are also affected by systemic inflammation in the lungs, which may also lead to poor postoperative outcomes.^[16]^ There may be other factors that worsen poor pulmonary conditions and postoperative respiratory status that cannot be measured and may magnify the small problem of low iCrs. We also reported the intervention that should be used for emergency patients, but the fact that the adverse effects of low iCrs were smaller in patients scheduled for surgery suggests that preoperative and intraoperative management has some significance.

This study had several limitations. First, iCrs was not measured using the standardized procedure. Throughout anesthesia, the tidal volume or PEEP was determined by attending anesthesiologists. As mask ventilation proceeded with tracheal intubation, one may suggest that some atelectasis would occur at the time of intubation. Second, we included various surgical patients, including those who had undergone lung surgery or laparotomy. These interventions may have caused a large decrease in Crs during surgery. Thus, understanding the clinical significance of hourly changes in Crs has become difficult. Third, there were large differences in patient demographics between the 2 groups. For example, we found that emergency hospital admissions were more frequent in the low Crs group. Even with clinically relevant adjustment for logistic regression analysis, we must be aware of the confounding factors related to outcomes. Fourth, we did not calculate the sample size because the study was retrospective. We may have overestimated small differences that were not clinically problematic.

## 5. Conclusions

The low Crs at the induction of anesthesia was associated with a smaller decrease in Crs and a higher chance of longer postoperative oxygen therapy duration and mechanical ventilation than in the normal to the high Crs group. These findings suggested that a decrease in a single parameter of Crs underestimates the goodness of perioperative respiratory management. Given that this relationship especially strengthened in emergency surgery, suitable respiratory management for emergency patients with low Crs should be investigated in the future.

## Acknowledgments

Assistance with the article: We appreciate Ms Eriko Ikeda (a health information manager at the University of Fukui Hospital) for extracting data from the electronic medical records and Mr Riku Adachi (University of Fukui) and Dr Takahiro Tokunaga (Toyama Hospital and University of Fukui) for supporting the statistical analyses.

## Author contributions

**Conceptualization:** Koji Hosokawa, Yukiko Yamazaki, Kenji Shigemi.

**Data curation:** Kayo Ishihara.

**Formal analysis:** Katsuya Tanaka.

**Investigation:** Katsuya Tanaka, Kayo Ishihara.

**Methodology:** Koji Hosokawa, Yuka Matsuki.

**Software:** Koji Hosokawa.

**Supervision:** Kenji Shigemi.

**Validation:** Koji Hosokawa.

**Visualization:** Katsuya Tanaka, Koji Hosokawa.

**Writing – original draft:** Katsuya Tanaka.

**Writing – review & editing:** Koji Hosokawa, Kayo Ishihara, Yukiko Yamazaki, Yuka Matsuki, Kenji Shigemi.

## Supplementary Material


