# Supplementary material for: Baseline respiratory system compliance and its decline during general anesthesia for surgery: A retrospective observational study
Source: Medicine (Baltimore). 2025 Jun 13;104(24):e42845. doi: 10.1097/MD.0000000000042845 (PMC12173264; doi:10.1097/MD.0000000000042845)
Supplement: Supplementary file 1 [file medi-104-e42845-s001.docx]

Supplemental document:

**Baseline respiratory system compliance and its decline during general anesthesia for surgery; a retrospective observational study**

Tanaka K., Hosokawa K., Ishihara K., Yamazaki Y., Matsuki Y., Shigemi K.

1. Anesthesia induction and respiratory settings

As a routine protocol, general anesthesia was induced using propofol (target control infusion, 2.0‒3.5 microg/ml) or midazolam (0.4‒1.2 mg/kg) and remifentanil (0.15‒0.3 microg/kg/min) with rocuronium (0.6‒1 mg/kg). Mask ventilation was continued until train-of-four counts reached 0 or 1. A minimal apneic interval was followed by tracheal intubation.

The respiratory setting was determined by admitted anesthetists. The volume-guaranteed pressure control ventilation (PCV-VG) were used as the routine respiratory mode on Aisys CS 2. Tidal volume of respiration was set 7‒10 × body weight (mL) and PEEP were set 5 cmH_2_O. We had not established a role that the recruitment maneuver was applied immediately after tracheal intubation.

Crs was displayed on Aisys CS 2 using manufacture-driven calculations. As the manufacture answered, the calculation formula is followed; in case of pressure control ventilation, the value of dynamic Crs used; Crs = tidal volume/(maximum pressure‒PEEP)]. Other respirator mode and parameters were also obtained and stored in GAIA anesthetic chart system. iCrs (Crs just after tracheal intubation while the induction of anesthesia was performed) was the mean of nine consecutive minute values after tracheal intubation.

2. Postoperative respiratory managements

The criteria that patients were not extubated from trachea in the operation theater were; high risk of postoperative bleeding (underwent cardio-pulmonary bypass, carotid artery surgery), larynx or mandibular surgery with plastic surgery, and delayed consciousness after cessation of sedatives. After certain periods of postoperative observation, tracheal extubation was decided with general clinical condition and no abnormal signs with spontaneous awaking and breathing trials.

Supplementary Table 1. Changes in respiratory parameters during anesthesia

|  |  | Intuba-tion | 1 h | 2 h | 3 h | 4 h | Extuba-tion |
| --- | --- | --- | --- | --- | --- | --- | --- |
| Tidal volume (mL) | All | 408 [358, 458] |  |  |  |  |  |
|  | Low iCrs | 372 [331, 419] | 376 [332, 422] | 377 [336, 422] | 379 [335, 423] | 384 [345, 424] | 368 [320, 421] |
|  | Other | 419 [371, 467] | 421 [377, 472] | 423 [379, 470] | 426 [380, 472] | 425 [379, 474] | 409 [359, 466] |
| Tidal volume/body weight (mL/kg) | All | 6.8 [6.2, 7.5] |  |  |  |  |  |
|  | Low iCrs | 6.6 [5.9, 7.4] | 6.8 [5.9, 7.5] | 6.7 [5.9, 7.4] | 6.6 [5.7, 7.4] | 7.0 [5.8, 7.4] | 6.5 [5.7, 7.4] |
|  | Other | 6.9 [6.3, 7.5] | 7.0 [6.4, 7.7] | 7.0 [6.4, 7.7] | 7.0 [6.4, 7.7] | 7.0 [6.3, 7.6] | 6.8 [6.1, 7.6] |
| Peak airway pressure (cmH₂O) | All | 14 [13, 16] |  |  |  |  |  |
|  | Low iCrs | 16 [15, 18] | 17 [15, 19] | 17 [15, 20] | 17 [15, 19] | 17 [15, 19] | 15 [14, 18] |
|  | Other | 14 [13, 15] | 15 [13, 17] | 15 [14, 17] | 15 [13, 17] | 15 [13, 17] | 14 [13, 16] |
| PEEP (cmH₂O) | All | 4.7 [4.1, 5.1] |  |  |  |  |  |
|  | Low iCrs | 4.6 [4.0, 5.1] | 5.0 [5.0, 6.0] | 5.0 [4.9, 6.0] | 5.0 [4.7, 6.0] | 5.0 [4.2, 5.9] | 3.9 [3.1, 4.8] |
|  | Other | 4.7 [4.2, 5.1] | 5.0 [5.0, 5.1] | 5.0 [4.9, 5.1] | 5.0 [4.9, 5.1] | 5.0 [4.8, 5.1] | 4.0 [3.2, 4.6] |
| Driving pressure (cmH₂O) | All | 7.8 [6.4, 9.4] |  |  |  |  |  |
|  | Low iCrs | 10.4 [9.0, 12.0] | 10.8 [9.5, 12.5] | 10.9 [9.4, 12.6] | 10.7 [9.3, 12.8] | 10.7 [9.2, 12.5] | 9.8 [8.5, 11.5] |
|  | Other | 7.2 [6.2, 8.4] | 8.6 [7.3, 10.1] | 8.8 [7.4, 10.5] | 8.8 [7.5, 10.5] | 8.8 [7.4, 10.4] | 8.0 [6.9, 9.5] |
| Crs (mL/cmH₂O) | All | 52.0 [42.8, 63.0] |  |  |  |  |  |
|  | Low iCrs | 37.0 [32.6, 40.1] | 35.4 [29.5, 40.7] | 34.7 [29.0, 40.2] | 34.8 [28.3, 40.3] | 35.4 [29.6, 41.3] | 36.5 [31.1, 42.3] |
|  | Other | 56.8 [50.0, 66.8] | 49.2 [41.3, 58.6] | 47.8 [39.9, 57.2] | 47.3 [39.2, 56.6] | 47.8 [39.2, 56.6] | 49.8 [42.5, 59.1] |
| Crs change from intubation (%) | All | 1 (ref.) |  |  |  |  |  |
|  | Low iCrs | 1 (ref.) | –2.4% [–14.4%, 10.7%] | –3.7% [–16.8%, 9.9%] | –4.5% [–19.2%, 10.8%] | –2.1% [–16.5%, 11.5%] | 0.7% [–12.5%, 16.0%] |
|  | Other | 1 (ref.) | –11.8% [–25.6%, –3.6%] | –15.0% [–28.7%, –6.1%] | –16.2% [–29.2%, –6.7%] | –16.6% [–29.3%, –6.0%] | –13.0% [–23.7%, –2.5%] |

Values are presented as percentages or medians [interquartile ranges]. Low iCrs was defined as < 43 mL/cmH_2_O of Crs at the induction of anesthesia. Crs, respiratory system compliance; PEEP, positive end-expiratory pressure; ref., reference.

Supplementary Table 2. Clinical outcome

|  | | All case (n=5,568) | Crs value at induction of anesthesia | | |
| --- | --- | --- | --- | --- | --- |
|  |  |  | < 43 mL/cmH_2_O (n=1,392) | ≥ 43 mL/cmH_2_O (n=4,176) | p value |
| Duration of postoperative oxygen therapy (day) | | 2 [1, 2] | 2 [1, 2] | 2 [1, 2] | 0.001 |
| Mechanical ventilation in the 28-day postoperative period | | 315 (5.7%) | 117 (8.4%) | 198 (4.7%) | 0.001 |
|  | Duration (day) | 4 [2, 7] | 4 [2, 9] | 4 [2, 7] | 0.078 |
| Admission to ICU | | 613 (11.0%) | 194 (13.9%) | 419 (10.0%) | 0.001 |
|  | Length of postoperative ICU stay (day) | 3 [2, 7] | 4 [2, 8] | 3 [2, 6] | 0.001 |
| Length of hospital stay (day) | | 13 [8, 22] | 15 [9, 23] | 12 [8, 21] | 0.001 |
| In-hospital mortality | | 22 (0.4%) | 12 (0.9%) | 10 (0.2%) | 0.003 |

Values are presented as numbers (percentages) or medians [interquartile ranges]. Crs, respiratory system compliance; ICU, intensive care unit.

Supplementary Table 3. Comparison of clinical outcomes, as incidence ratio, between the two Crs groups.

|  | | Adjusted Odds ratio  (95% confidence interval) | p value |
| --- | --- | --- | --- |
|  |  |  |  |
|  |  |  |  |
| Postoperative oxygen therapy (≥3 days) | | 1.60 (1.29 to 1.97) | 0.001 |
| Mechanical ventilation during the 28-day postoperative period | | 1.87 (1.33 to 2.63) | 0.001 |
|  | Mechanical ventilation following surgery (≥4 days) | 1.55 (1.02 to 2.36) | 0.039 |
| Admission to ICU | | 1.30 (0.98 to 1.73) | 0.067 |
|  | ICU stay (≥6 days) | 1.54 (1.05 to 2.26) | 0.028 |
| Hospital stay (≥21 days) | | 1.05 (0.88 to 1.24) | 0.585 |
| In-hospital mortality | | 2.23 (0.85 to 5.84) | 0.101 |

Logistic regression analysis was conducted to show the odds ratio adjusted by age, American Society of Anesthesiologist physical status, duration of anesthesia, and surgical categories. Crs, respiratory system compliance; ICU, intensive care unit.

Supplementary Figure 1. Duration of postoperative oxygen therapy between the two respiratory system compliance groups.


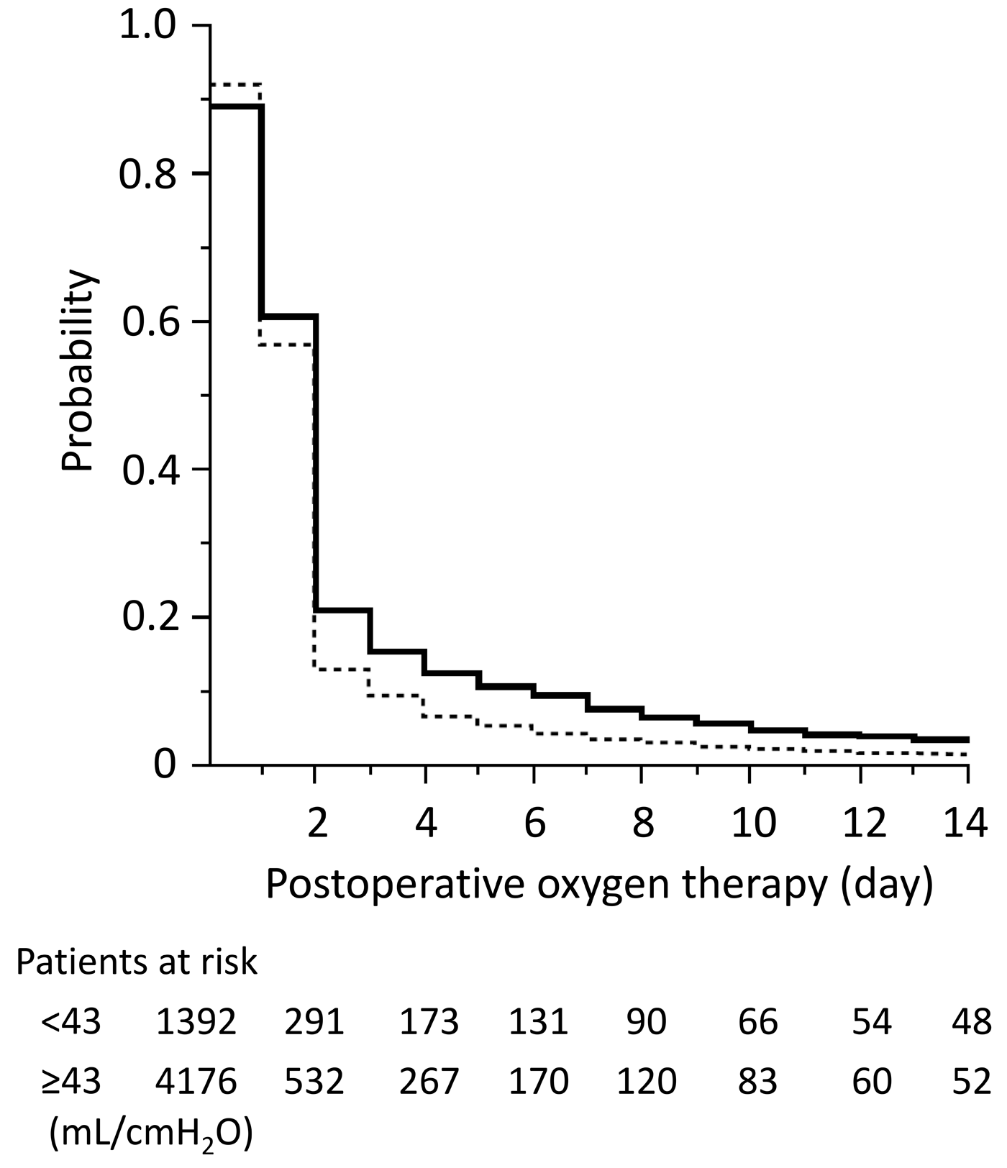


Low iCrs was defined as < 43 mL/cmH_2_O of Crs at the induction of anesthesia. The probability of oxygen therapy is shown in low iCrs group (bold line) and the other group (dashed lines). The Kaplan-Meier curves shows that the groups are statistically different (Log rank, p<0.001). Crs, respiratory system compliance.
